# Evaluating Individual Differences in Implicit Perceptual-Motor Learning: A Parallel Assessments Approach

**DOI:** 10.3390/jintelligence13090115

**Published:** 2025-09-08

**Authors:** Y. Catherine Han, Kelsey R. Thompson, Paul J. Reber

**Affiliations:** 1 Department of Medical Social Sciences, Feinberg School of Medicine, Northwestern University, Chicago, IL 60611, USA; ych@northwestern.edu; 2 Office of Institutional Effectiveness and Assessment, St. Olaf College, Northfield, MN 55057, USA; thomps22@stolaf.edu; 3 Department of Psychology, Northwestern University, Evanston, IL 60208, USA

**Keywords:** implicit learning, perceptual-motor sequence learning, interindividual differences, rank-order stability

## Abstract

Implicit learning describes learning from experience that is not available to conscious awareness. The question of whether some individuals are better implicit learners than others has suggested and may contribute to difference in performance among experts. Across four experiments, adult participants completed the Serial Interception Sequence Learning (SISL) task across multiple parallel learning assessment forms. Previously, SISL sequence-specific performance has been shown to resist explicit knowledge influence, allowing for repeated reassessments of implicit learning with novel statistical structure. Our findings indicate that group-level sequence-specific performance occurred robustly in each reassessment; however, participants who exhibited more sequence-specific performance on one assessment did not exhibit better performance on parallel assessments, indicating no rank-order stability in learning. In all four experiments, with two to twelve reassessments of learning, no participants exhibited consistently better sequence learning rates than the other participants, indicating no evidence for a better ability in implicit learning. Measurements of other cognitive constructs, such as processing speed collected in parallel, exhibited robust individual differences. In Experiment 4, a general battery of cognitive measurements showed typical individual differences in measures of working memory, processing speed, and personality, but none correlated with implicit learning ability. We hypothesize that implicit learning arises from a general process of neuroplasticity reorganizing functions during practice and that our findings suggest that this process occurs at a basically similar rate across all people. Everybody learns from practice implicitly, but results suggest that the learning rate does not vary substantially across this sample.

## 1. Introduction

Research has established that individuals differ in cognitive abilities in meaningful, consistent ways (Carroll 1993). These abilities span several domains, including but not limited to working memory capacity (Conway et al. 2003; Kane and Engle 2002), processing speed (Sheppard and Vernon 2008), visuospatial cognition (Hegarty and Waller 2005), and fluid intelligence (Salthouse et al. 2008; Salthouse and Pink 2008). Individuals who perform better in one cognitive domain tend to perform well in others, and this pattern, described as the *positive manifold*, is the idea that different cognitive abilities are positively intercorrelated (Spearman 1904). This relationship among cognitive ability measures has been used to support the idea of an underlying latent general intelligence factor *g*, where *g* represents a broad, domain-general cognitive ability that contributes to performance across diverse tasks.

Embedded in this idea is that most cognitive abilities are derived from this factor and that there are few stable cognitive abilities that are independent of *g*. However, recent advances in cognitive neuroscience complicate this perspective by identifying functionally and anatomically distinct neural regions or networks for different cognitive functions (McFarland 2017). For example, memory systems research has shown that the neural basis of memory acquisition relies on specific neural systems, the medial temporal lobe (Squire 2009), in a manner that is at least somewhat independent of other high-level cognitive functions (Janowsky et al. 1989; Wheeler and Stuss 2003). This raises the possibility that there may be cognitive abilities that are at least partially independent of general intelligence.

Memory is likely to be an important factor in any understanding of differences in adult cognitive ability, as differences in memory function likely have a significant influence on the impact of education and other learning experiences. As a result, score differences on tests designed to measure cognitive abilities in adults may be difficult to interpret without accounting for individual differences in memory across the lifespan. This challenge is further complicated by the concept of *implicit learning*, a form of memory that has been discussed to operate outside of conscious awareness and differs from traditional views of declarative or explicit memory that is based more on facts and events. Implicit learning has been hypothesized to reflect a general plasticity mechanism by which neural systems reorganize based on experience or practice (Reber 2013). It has been argued that interindividual differences in performance on cognitive measures related to *g* may reflect differences in plasticity function (Garlick 2002), and general intelligence measures may be strongly influenced by how well individuals can learn and retain information (Carroll 1997).

Consideration of the contribution of learning systems to general cognitive function has directed attention to the phenomenon of implicit learning (Reber 1967, 1989) and whether expression of this type of learning exhibits stable interindividual differences across people (Kalra et al. 2019; Kaufman et al. 2010). Implicit learning was originally defined as a type of memory that operates largely outside of awareness (Reber 1967, 1989). Neuropsychological studies subsequently established that this type of learning does not depend on the medial temporal lobe memory system that supports the learning of explicit factual and event related information (Squire 1992).

Implicit learning has been reported to occur across a wide range of tasks spanning visual, motor, linguistic, and cognitive domains. This observation has led to the proposal that a universal neuroplasticity mechanism supports implicit learning (Han et al. 2022; Reber 2013), rather than conceptualizing this type of memory as dependent on a single neural system as explicit memory is. As such, this approach characterizes implicit learning as an emergent phenomenon in which improved processing occurs via experience-dependent neural reorganization during repeated experience. If this process operates more effectively in some people, there would be substantial trait-level differences across a range of functions that are improved by repetitive practice. From this view of memory systems, we might hypothesize that implicit and explicit memory will correlate differently with cognitive assessments that contribute to the broad cognitive positive manifold and potentially show different tendencies towards individual differences across the population.

The question of whether implicit learning represents a stable, individual cognitive trait and whether it relates to general intelligence has been explored in several previous studies. Given the wide array of putative measures of implicit learning, it is unsurprising that there are mixed findings. Early examination of this using the Artificial Grammar Learning (AGL) task found no reliable relationship between implicit learning and performance on other measures that traditionally correlated with *g* (Reber et al. 1991). This result was supported by several subsequent studies (Danner et al. 2017; Gebauer and Mackintosh 2007; McGeorge et al. 1997). In contrast, studies using another popular implicit learning task, the Serial Reaction Time (SRT) task, have demonstrated modest associations with some components of psychometric intelligence, like processing speed, matrix reasoning, and verbal analogical reasoning (Kaufman et al. 2010; Sobkow et al. 2018).

The SRT task is a frequently studied perceptual-motor sequence-based measure of implicit learning that has been shown to relate to self-reported personality components like openness (Kaufman et al. 2010; Sobkow et al. 2018) and to correlate with real-world outcomes like academic performance in language and math (Kaufman et al. 2010; Pretz et al. 2010). Yet, findings about cross-task correlations with SRT are inconsistent. Pretz et al. (2010) reported that SRT, but not AGL performance, was related to standardized Math and English test scores. However, Siegelman and Frost (2015) found that SRT performance did not relate to statistical learning performance or performance on other cognitive tasks measuring fluid intelligence, working memory capacity, and executive functioning.

Other studies that have looked at implicit learning across tasks have identified moderate cross-task correlations, including SRT, probabilistic classification, and implicit category learning (but not AGL) (Kalra et al. 2019). Measures from these tasks appear to be related to each other but not measures of working memory or IQ. Other studies have reported very weak cross-task correlations among implicit learning measures, such as between SRT, the Alternating Serial Reaction Time (ASRT), and the Visuomotor Adaptation (VMA) tasks (Stark-Inbar et al. 2016) or SRT and AGL (Sobkow et al. 2018).

The complexity and inconsistency of prior attempts to examine individual differences in implicit learning leave open three related questions. First, is there a stable, trait-like implicit learning ability that leads some people to simply learn faster from repetitions than others? Second, is this ability related to other measures of cognition, such as working memory capacity and processing speed, that have been hypothesized to be related to general fluid intelligence? Third, is there a common underlying mechanism supporting individual ability that contributes to all phenomena that have been associated with implicit learning?

A fundamental methodological challenge in answering these questions is that it has been commonly observed that implicit learning protocols with cognitively healthy participants often suffer from the problem of explicit contamination (Dulany et al. 1984; Frensch and Miner 1994; Willingham et al. 1993). Even though the tasks are designed to observe and measure learning outside awareness, some participants may explicitly, consciously infer key elements of the embedded statistical structure. The ability to deduce hidden elements of a task is very likely to reflect a process dependent on working memory and is very plausibly the reason why the relationship between implicit learning and working memory is well-described as “complicated” (Janacsek and Nemeth 2013; Martini et al. 2015). This idea was studied directly by Gebauer and Mackintosh (2007), who found low correlations between implicit learning task performance under standard learning conditions (AGL, sequence learning, process control) but higher correlations when participants were given instructions that encouraged use of explicit strategies. The potential multiplicity of strategic approaches to putatively implicit learning tasks has been debated extensively in the literature (Dienes and Berry 1997) and described as the challenge to obtain a “process pure” measure of implicit learning resistant to explicit influence.

**Figure 1:**
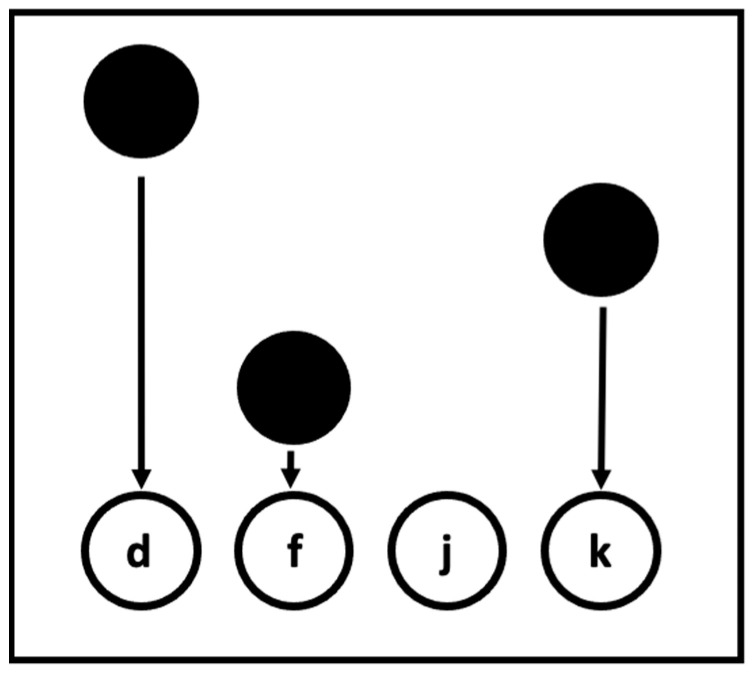
The Serial Interception Sequence-Learning (SISL) task. Circular cues (shown black) travel vertically down the computer screen towards target zones (open circles) that are associated with keyboard keys (‘d’, ‘f’, ‘j’, ‘k’). Participants attempt to press the corresponding key precisely when the traveling cue moves through the bottom target zone (here in the order of f, k, then d). Participants are not told that the sequence of cues follows an embedded 12-item repeating sequence. Sequence knowledge is measured as the increased accuracy in performance during the repeating sequence compared with periods where the cue follows an unfamiliar sequence (Sequence-Specific Performance Advantage; SSPA).

In the studies presented here, we will focus on the first two of the three questions raised above by capitalizing on a relatively newer implicit learning paradigm that exhibits much better process purity than most previously studied tasks, the Serial Interception Sequence Learning (SISL) task (see Figure 1). The SISL task is based on perceptual-motor sequence learning, like the well-studied SRT (Nissen and Bullemer 1987) or ASRT tasks, with the key difference that instead of using a sequence of choice reaction time responses, it uses a sequence of interception responses to moving cues. The SISL task shows relatively low correlations between measures of implicit and explicit learning (Sanchez et al. 2010), but, more importantly, the core measure of implicit learning has been shown to be unaffected by the existence of parallel explicit sequence knowledge (Sanchez and Reber 2013). Likely due to the fact that the SISL task depends on precisely timed responses to rapidly moving cues, full explicit knowledge provided by memorization before practice did not increase task performance. In general, for a choice reaction time task, conscious knowledge of the next cue will lead to very short (even negative) reaction times, although with the relatively more complex structure of the ASRT task, Song et al. (2007) reported a similar resistance to explicit contamination.

The problem that most prior measures of implicit learning are affected by explicit contamination is likely the reason why previous attempts to measure correlations with implicit learning have not used repeated assessments with the same implicit learning task. With the SISL task, we demonstrate here the utility of a *parallel forms* reliability approach, in which the learning process is measured repeatedly with novel material on each assessment. Prior attempts at measuring stable differences in implicit learning depended on multiple implicit learning tasks (e.g., Kalra et al. 2019), but the frequent risk of explicit contamination could create spurious cross-task correlations unrelated to implicit learning. The inability to remeasure the learning process means there is very little evidence of stability or reliability in implicit learning measurements. Prior studies demonstrating test–retest reliability (Kaufman et al. 2010; Oliveira et al. 2024; Salthouse et al. 1999; Siegelman and Frost 2015; Stark-Inbar et al. 2016; Kalra et al. 2019; West et al. 2021) relied on remeasuring acquired learning, not reassessing the learning rate with novel material. For a person to have a superior (or inferior) implicit learning ability, they should demonstrate consistently better learning each time they encounter an opportunity to improve performance from extraction of embedded statistical structure. With the SISL task, we are able to observe repeated learning processes with a novel repeated sequence on each assessment with multiple assessments within (Experiments 1–3) and across days (Experiment 4).

Most studies using the SRT or ASRT task assess implicit perceptual-motor sequence learning using a fixed set of materials such that all participants learn the same sequence (Nissen and Bullemer 1987; Howard and Howard 1997). With the SISL task, each participant is assigned a random 12-item sequence from a set of 256 possible frequency-balanced sequences that is practiced and eventually shows a sequence-specific benefit in accurate responding. By changing the underlying repeating sequence, the learning process is reset and can be re-observed for this novel material. In the experiments presented here, we demonstrate that this process can be carried out 2, 4, or 12 times to create a comparable set of parallel forms for repeated reassessment of the learning rate for this process pure implicit learning task.

Through this methodology, the set of studies presented here tests whether we can observe the ability trait-like differences in implicit learning ability, i.e., whether a participant who is a better implicit learner than average should exhibit better implicit learning performance consistently across each assessment. Stable rank-order correlation in implicit learning scores across forms would suggest the presence of individual differences in implicit learning ability, raising the possibility that such an ability could group together with domain-general cognitive functioning, or *g*.

Thus, the aims of the current studies are to (1) assess the stability of implicit learning performance across multiple administrations of the SISL task using different sequences for each re-administration, (2) determine whether consistent performance reflects a stable trait, and (3) determine whether this trait is linked to other forms of cognitive ability related to *g.* The implications of this work extend our current understanding of whether implicit learning processes, typically assumed to be unconscious and automatic, systematically differ across individuals in a way that mirrors more explicit memory and other cognitive abilities. Through the use of a parallel-forms approach not commonly found in other studies of implicit learning individual differences, our study designs present a psychometric advancement over prior work that has assessed repeated performance of the same implicitly learned content, making it possible to disentangle “true” learning ability from task familiarity or learning-to-learn effects.

## 2. Materials and Methods

### 2.1. Serial Interception Sequence Learning (SISL) Task

In the SISL task, circular cues move vertically down a screen towards one of four labeled target zones (Figure 1). Participants are instructed to press the corresponding key on a keyboard (D, F, J, or K; Figure 1) as precisely as possible the moment the cue moves through the bottom target zone. The task layout is presented on a typical computer monitor within the laboratory (23”, 1920 × 1080 resolution) within a frame of 600 × 800 pixels (~60 cm typical viewing distance, ~20.0° visual angle for typical viewing distance). Cues (90 pixels in diameter, 2.38 cm, 2.27° visual angle) appeared at the top of the display and moved vertically downwards towards one of four targets, 565 pixels away vertically, and spaced 200 pixels (5.29 cm, 5.05° visual angle) apart horizontally across the bottom of the screen. The travel time for the cues from appearance to reaching the target zone was 1.5 s at the initial presentation of the task, reflecting a cue velocity of 297 pixels/s or 7.86 cm/s (based on a 96-pixel-per-inch monitor resolution) from onset to the target zone on the screen. Responses were considered correct if the key was pressed within approximately 160 pixels (4.23 cm) of the moment of perfect overlap. A response was scored as correct whenever a cue was within this distance even if other cues might also be approaching the moment of overlap (as the task speeds up, see below). After a response, feedback was provided by the target circle corresponding to the keypress, which flashed green for correct responses and red for incorrect responses. In addition, the cue was removed from the screen for correct responses to make it clear to the participant that the response to that cue was made successfully. For scoring purposes, a single cue and keyboard response is defined as one “trial.”

Participants were not told that the cues contained an embedded, 12-item, repeating sequence. In each administration of the SISL task, the embedded repeating sequence for each participant was randomly selected from the set of 256 possible 12-item second-order conditional (SOC) sequences (Reed and Johnson 1994) that can be constructed using four different response locations. In a SOC sequence, each of the four response locations occurs equally often (three times) and sequentially, with no response repetitions. Every other pairwise combination occurs exactly once (e.g., ‘DF’, ‘DK’, ‘DJ’, etc.). With this structure, it is not possible to predict the next item in the 12-item sequence based on frequency or simple transitional frequency. However, the second-order transitional frequency (or trigram frequency) is fully predictive of the next item in the sequence. Learning the transitional frequency will support improved performance on a practiced repeating sequence.

The repeating sequence further included an embedded inter-cue timing structure with 6 long and 6 short intervals between cues, e.g., ‘K_S_F_S_J_L_D_S_K_L_D_L_F_S_K_L_J_S_F_S_D_L_J_L_’, where S = short inter-cue timing (initially 250 ms), L = long inter-cue timing (initially 500 ms). The inter-cue onset time was less than the onset to target travel time, meaning that there are generally multiple cues moving simultaneously on the screen at any given moment. The assignment of the inter-cue timing structure was unique and randomly assigned for each participant but remained consistent for that individual’s assessment (i.e., the order and timing of the sequence was the same for training and test).

The primary measure of sequence learning is the increased accuracy during task performance when the cues follow the practiced repeating sequence compared with periods where the cue order does not. The Sequence Specific Performance Advantage (SSPA) is calculated as a subtraction score of response accuracy (percent correct) for a series of trials within the practiced repeating sequence compared with accuracy during novel repeating sequences (% correct repeating trained sequence − % correct untrained sequences). SSPA is reported in units of percent correct (%). In each of the SISL protocols used across the experiments reported here, participants complete an initial training phase that primarily contains repetitions of a sequence to be learned, followed by a test phase that contains repetitions of that sequence and blocks of repetitions of two foil sequences (also with SOC structure). SSPA is also calculated at test as the differential accuracy between the trained and untrained sequences. Positive scores reflect better performance on the trained sequence that can only be due to the learner’s experience with that sequence prior to the test. Chance performance on this measure is 0%, reflecting no difference in accuracy and no sequence-specific learning.

The SSPA difference score relies on overall task performance being neither at ceiling nor at floor for the task. To ensure this, an individually adaptive speed adjustment algorithm is used to target an overall performance level of 80% throughout the task. If participants are performing near ceiling (100% overall accuracy), the overall cue speed is increased, which leads to a decrease in accuracy. Likewise, if overall performance is less than 75% correct, the overall cue speed is decreased, allowing for more accurate responding. These adjustments are calculated for each individual participant dynamically during task performance. The speed algorithm is evaluated every 12 trials and is performance-adaptive. If 11 or 12 responses are correct (>91.7%), the cue speed is increased by ~5%. If 9 or fewer responses were correct (<75%), the cue speed is decreased by ~5%. Because these adjustments occur at the level of each sequence (12 items) and regardless of whether the sequence type is trained or untrained, performance on either trained or untrained sequences should not be selectively influenced by the speed algorithm.

Inter-cue timing intervals are scaled with task speed to maintain a constant relative ratio of the long and short inter-cue intervals. Speed was adaptive during both training and test phases of each assessment. As a result of this adaptive approach, overall task accuracy is generally constant across participants and does not indicate any individual differences in aptitude for the SISL task. However, the speed at which the adaptive algorithm settles to produce an overall accuracy of 80% becomes the effective measure of task ability.

Throughout the experiments reported here, two key measures of participant performance on SISL are used as the dependent variables that may express stable trait-like individual differences in performance: SSPA and Speed. The SSPA measure reflects the participants’ learning of the repeating sequence, with larger scores indicating greater learning. As found in prior work (Sanchez and Reber 2012), SSPA increases linearly as a function of log-linear repeating sequence repetition amount. The Speed measure reflects overall ability with the SISL task, independent of any sequence-specific effects.

### 2.2. Participant Exclusion Criteria

While the game-like nature of the SISL task generally produces good compliance with instructions, its rapid pace means that non-compliance or periods of inattention can quickly result in a high number of erroneous responses, distorting measures of learning. To avoid inflating variance, which would weaken the estimation of stable trait-like individual differences, data exhibiting characteristics of non-compliance were excluded from analyses. Performance was evaluated in 180-trial blocks, regardless of sequence type. Participants were excluded if they met any of the following criteria: (a) more than 50% of trials missed, suggesting inattention, (b) more than 270 keystrokes recorded, indicating “button mashing,” or indiscriminate and repeated keypresses rather than accurate, precisely timed responses, or (c) an overall accuracy below 25%, in spite of the adaptive speed algorithm intended to maintain performance near 80%.

Across studies, adult participants were recruited from three sources: the Northwestern University introductory psychology participant pool, Amazon Mechanical Turk, or local recruitment from the Chicago area. All participants completed the studies online. The same exclusionary criteria were used regardless of participant recruitment method. While participant demographic information was not collected (e.g., age, gender, race/ethnicity), participants were required to be 18 years or older, consent to their enrollment in the study in English, and be able to comprehend the study instructions, which were provided in English. Exclusion rates were 20–25% across experiments and were reported in each experiment.

### 2.3. General Analytical Approach

All analyses were conducted using R (R Core Team 2024). Each of the experiments reported here examines within-session sequence learning using SISL and a parallel-forms reliability method, in which participants learned multiple unique, repeating SOC sequences within the same one-hour session. For both SSPA (sequence-specific knowledge) and Speed (sequence-general knowledge), learning effects across each unique SOC sequence assessment will be assessed via one-way ANOVAs with linear contrasts. Null effects will be further explored using Bayes Factor analyses, which will provide further information regarding the likelihood of differences between groups. For SSPA, group-level learning for each sequence assessment will be assessed via one-sample *t*-tests against *mu* = 0, which is chance performance (Kass and Raftery 1995).

To assess the rank-order stability of performance across unique sequences, Spearman rho (*ρ*) correlations were also used. Better learners should produce consistently better learning scores across assessments, leading to persistent correlations between the parallel-forms measures. To further assess the stability of individual differences, we examined the average-rater reliability intraclass correlations (ICCs) across the number of k assessments per participant (2–4 sequence assessments) using a two-way random model (ICC(2,k)), which provides a measure of absolute agreement between raters, including any systematic differences between them, and random residual errors between sequence assessments (Liljequist et al. 2019; McGraw and Wong 1996; Shrout and Fleiss 1979). We also report consistency ICC (ICC(3,k)) using an average-rater, two-way mixed model, which places greater emphasis on rank-order consistency and is more sensitive to systematic shifts in means across participants. ICCs also provide information about the proportion of variability attributed to interindividual differences or “true” trait levels between individuals. More recent qualitative interpretation of ICCs suggest that ICCs < 0.5 are typically categorized as “poor”, 0.5–0.75 “moderate”, 0.75–0.9 “good”, and ICC > 0.90 “excellent” reliability (Koo and Li 2016). In contrast, small ICCs could indicate large heterogeneity among individuals, or high within-person and low between-person variability, particularly when paired with evidence of reliable group-level learning, which indicates that performance on the task is sensitive to some variation and is less attributable to just measurement error. These approaches are intended to provide a quantitative measure to identify individuals who exhibit consistently faster learning across the parallel form reassessments of the amount learned on each new SISL sequence. High ICC scores and high Spearman rank correlations will indicate that participants who score relatively higher on learning for one sequence consistently score higher on subsequent measures.

Importantly, we examine how much sequence-specific knowledge was learned via SSPA during the test phase only, as the test better isolates knowledge of the practiced repeating sequence compared with training. Training involves repetitive training on the sequence for 80% of the block, whereas two novel repeating sequences are introduced at test, resulting in performance of the trained sequence at test for only 33% of the block. The goal of changing the composition of the test block was to reduce explicit knowledge contamination during the assessment of implicit knowledge acquired by requiring contrasting repetitions of a practice sequence with repetitions of an unpracticed sequence with the same structure. Thus, SSPA at test can only reflect knowledge of the practiced sequence, as confounds such as whether repeating or random sequences are used and repetition frequency are controlled for, which were issues in early SRT research.

### 2.4. Sample Size Justification

We began first by estimating the sample size that would be sufficient to detect known group differences. In three published works with visually cued SISL, we observed sequence-specific performance advantage effects of a SSPA = 10.1–16.3%, *SD* = 7.6–10.5%, Cohen’s *d* > 1.0 (Sanchez et al. 2015; Sanchez and Reber 2013; Thompson et al. 2014). We estimated that we would have >95% power to detect a reliable learning effect with a sample of *n* = 30 participants. Since the expectation that there would be a lack of stable individual differences is essentially a null hypothesis, we conducted a power analysis to detect a weak but significant ICC of 0.4, assuming the H_0_ ICC would be 0 (that there is no systematic variance between participants beyond chance) (Rathbone et al. 2015; Zou 2012). We estimated that we would have 80% power to detect a significant moderate ICC with *n* = 45. However, we oversampled in anticipation that the effect size would be smaller than projected and also to minimize sampling error.

## 3. Experiment 1

### 3.1. Participants

In Experiment 1, *n* = 79 participants were recruited through Amazon’s Mechanical Turk online interface (demographics unavailable), and 19 of these were subsequently excluded (24%), leaving 60 participants’ data for analysis. Of the 19 excluded, 9 participants had primarily missed responses, 4 had primarily excess responses, and 6 had primarily non-compliant levels of accuracy.

### 3.2. Procedure

**Figure 2:**
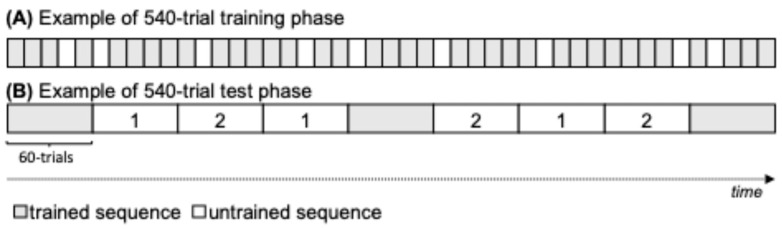
An example schematic of the SISL training and test phase structure. (**A**) During training, participants are exposed to a 12-item repeating sequence 80% of the time randomly intermixed with a novel untrained sequence. (**B**) During the test phase, participants complete longer 60-trial blocks of the trained sequence randomly intermixed with 60-trial blocks of one of two novel repeating sequences (labeled 1 and 2, accordingly, in the above schematic).

Participants completed four parallel-form SISL task assessments in succession with a different embedded repeating sequence in each assessment during a 1 h session, as described in General Methods (Section 2). Within each assessment, participants completed a 540-trial training phase followed by a 540-trial test phase. The training phase consisted of 36 repetitions of the embedded repeating sequence (432 trials) with 108 trials of non-repeating segments interspersed between repetitions. The training phase was constructed as nine sub-blocks of 60 trials that contained four sequence repetitions and 12 cue location trials taken from a novel SOC sequence (Figure 2). The 80% sequence rate has been previously found to produce robust learning with relatively low levels of explicit sequence recognition (Sanchez and Reber 2012). The test phase (540 trials total) consisted of 180 trials of the practiced sequence (33% of test) and 360 trials of two novel repeating sequences (180 trials each, 67% of test). The practiced sequence and novel repeating sequences were each subdivided into 60-trial blocks (three 60-trial sub-blocks each for the practiced sequence, novel repeating sequence 1, and novel repeating sequence 2), and the presentation of the practiced sequence and novel repeating sequence sub-blocks were randomly intermixed (Figure 2).

The SSPA measure of sequence learning was calculated as the difference in accuracy between the sub-blocks containing the trained repeating sequence and accuracy during repetitions of novel sequences (foils). Since the sequence and foils occur and repeat at the same rate during the test, the only source of improved performance for the practiced sequence is the learning that occurred during the training phase prior to the test. The adaptive task speed persisted across assessments, meaning each assessment started at the same speed at which the previous ended. Within the experimental session, participants went through four successive training and testing phases, with each assessment embedding a different novel repeating SOC sequence, so that learning began anew with each training phase. Four independent sequence-specific learning measures (SSPA) were collected alongside the four measures of overall task ability (Speed).

### 3.3. Results

**Figure 3:**
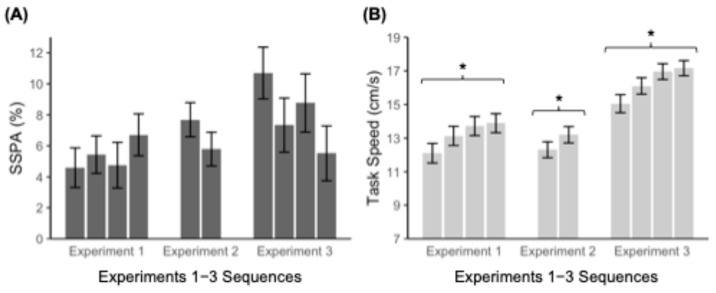
(**A**) Experiments 1 (*n* = 60), 2 (*n* = 60), and 3 (*n* = 51) Sequence-Specific Performance Advantage (SSPA) means across novel, independent sequence learning assessments with different sequences within-subject and across experiments. All sequences were learned (SSPAs > 0) but no reliable differences across sequences within subject were observed, suggesting no learning-to-learn or fatigue effects. (**B**) Experiments 1–3 task speed across the sequence assessments exhibited a reliable, significant increase (indicated by asterisk) in the adaptive task speed needed to keep overall performance at ~80% correct, reflecting increasing non-sequence-specific task ability over the sequence-learning measures within the experiment. For all measures, brackets reflect the standard error of the means.

Participants learned each of the four sequences reliably (Figure 3A), consistently exhibiting SSPA measures reliably greater than zero (overall *M* = 5.4%, *SE* = 0.7%, *ts* > 3.1, *ps* < .01). See Table A1 for further information. There was no evidence for either interference or learning-to-learn effects across the four assessments, *F*(1, 59) = 0.95, *p* = .33, *η*^2^ = 0.016 (see Figure 3 below). A Bayes Factor analysis was additionally calculated for null effects (*BF* = 0.22). Specifically, the data were approximately 0.22 to 1 in favor of the alternate hypothesis over the null, which is usually considered weak evidence for observable differences between groups (BF > 3 for evidence against H_0_) (Kass and Raftery 1995).

Spearman (rank-order) rho (*ρ*) correlations between SSPA were generally very low, *ρ* = .05 on average (averaging calculated by transformation to z score), with a range of *ρs*[−0.04, 0.29], *ps* > 0.14 (*p*-values corrected for multiple tests) across the six possible pair-wise correlations, except one significant correlation between the first and third sequence tests (*r* = 0.29, *p* = .02). This indicated that participants who performed better or worse on a particular sequence test did not do so consistently. Absolute agreement ICCs (2,k) and consistency ICCs (3,k) were calculated for SSPA, which indicated ICCs being close to 0 (absolute agreement ICC = 0.17, consistency ICC = 0.17).

In contrast, the same approach using the Speed score from each session’s performance (the individually adaptive speed at which the participant achieved 80% correct during training) produced highly reliable correlations across the four assessments. Spearman correlations in Speed were found to be highly reliable, *ρ* = 0.91 on average (*ps* < .001, *p*-values corrected for multiple tests). ICCs indicated that individual differences in Speed were “substantially” stable (absolute agreement ICC = 0.98, consistency ICC = 0.99). Participants who were generally faster on one assessment were consistently faster on all the assessments, indicating robust consistent individual differences in Speed. The average task Speed increased consistently across assessments, from *M* = 12.10 cm/s, *SE* = 0.59 cm/s to *M* = 13.89 cm/s, *SE* = 0.56 cm/s, *F*(1, 59) = 67.7, *p* < .001, *η*^2^ = 0.53 (Figure 3B), indicating that this measure of general task learning (not sequence-specific) persisted across assessments (but this consistent increase does not affect the rank correlation measure). In spite of consistent differences between SSPA and Speed, overall Speed level was weakly correlated with the averaged sequence learning SSPA measure, Pearson *r*(58) = 0.24, *p* = .07, *BF* = 1.35.

### 3.4. Experiment 1 Discussion

Participants learned four unique sequences presented in succession within the one-hour session and exhibited robust group-level learning of all four sequences. The lack of order effects suggested that each assessment provided an independent measure of the participant’s sequence learning that was unaffected by the other just-completed learning assessments. Although learning was consistent and robust, there was no evidence observed for stable rank-order differences in sequence-specific performance across participants. That is, participants who scored well on one assessment did not tend to consistently score well across all the assessments, suggesting minimal trait differences in sequence-specific knowledge. Of note, this was not a traditional measure of test–retest reliability for SISL, as we did not carry out two successive assessments for the same sequence. Instead, this approach provided multiple assessments of a potential underlying sequence learning ability across the four independent parallel forms. In contrast, the simultaneously collected Speed measure of general task ability (not sequence-specific) showed robust consistent individual differences across assessments. Participants who were generally “fast” (i.e., required a high adaptive cue velocity to keep performance at 80% correct) on one assessment tended to be consistently fast on all the assessments.

To validate the reliability and consistency of findings from Experiment 1, we conducted two additional studies that replicated the key findings using different participant samples and slight changes to the task. Experiments 2 and 3 are reported in full in Appendix A and Appendix B, respectively. In Experiments 2 and 3, participants again demonstrated reliable sequence-specific learning across multiple assessments, and there was no evidence of stable individual differences in sequence-specific performance across parallel assessments, indicating a lack of stable, trait-like stability in implicit learning ability. Although test–retest reliability within a given sequence (Experiment 2) was moderate, correlations across sequence assessments were notably low, emphasizing that trait-level differences in SISL sequence-specific learning are relatively small. Experiment 3 additionally adjusted the speed algorithm and test structure to rule out any potential methodological limitations influencing low individual differences in SSPA but found no improvements in individual stability of performance. By contrast and similar to Experiment 1, Speed, reflecting sequence-general performance, remained highly stable across assessments, reinforcing its robustness as an individual difference metric. 

## 4. Experiment 4

In addition to increasing the sample size and number of parallel-form SISL assessments, we incorporated a criterion validation method of assessing implicit learning individual differences by examining the relationships between SISL SSPA, SISL Speed, and a battery of psychological assessments. The measures chosen were selected because they were well-established measures of a construct, easily deployable for online data collection, or measures used in other implicit learning individual differences studies (e.g., Kaufman et al. 2010; Siegelman and Frost 2015). We employed factor analysis methods to identify whether implicit learning and other tests shared a common underlying construct. If the variance among individuals could consistently be accounted for by a particular factor, this would have provided evidence for the existence of a particular trait that reliably differed across people.

There are several measures of fluid intelligence and working memory capacity that have a long history within individual differences research (Conway and Kovacs 2013). Two of these measures, the Raven’s Progressive Matrices task (Raven et al. 1977) and the Operation Span task (Turner and Engle 1989), were adapted for online data collection here. Some have suggested working memory is essential for implicit learning, particularly when the attentional requirements for sequence learning are high under intentional conditions (Frensch and Miner 1994; Stadler 1995). However, prior individual differences research has shown implicit learning to not correlate well with other measures of working memory capacity (Feldman et al. 1995; Kaufman et al. 2010; Siegelman and Frost 2015) or explicit knowledge in general (McGeorge et al. 1997; Reber et al. 1991) except under slower, intentional learning conditions (Unsworth and Engle 2005) or when given an explicit rule discovery strategy (Gebauer and Mackintosh 2007). Additionally, implicit learning has generally been shown to not relate to measures of fluid intelligence like Raven’s Progressive Matrices (Kaufman et al. 2010; Siegelman and Frost 2015), though see (Sobkow et al. 2018), indicating that implicit learning, as measured by SRT, does not share a common mechanism with either working memory capacity or fluid intelligence constructs.

Kaufman et al. (2010) found SRT implicit learning to relate to processing speed, as measured by Speed of Information Processing sub-tests (figural speed) from the British Ability Scales. Similarly, Salthouse et al. (1999) found a significant correlation between SRT implicit learning and processing speed, as measured by the digit–digit and digit–symbol substitution (reaction time) tasks, which are also some of the most widely used tasks to describe young and older adult performance in the aging cognition literature (Hoyer et al. 2004). Here, we employed two different measures of processing speed found in Kaufman et al. (2010) and Salthouse et al. (1999) to test whether the implicit measure SISL SSPA related to these measures of processing speed. Compared to the prior SRT literature, which relies on reaction time as the dependent measure, performance on processing speed tasks may not relate to SISL SSPA, an accuracy-based measure of implicit learning. However, performance on processing speed tasks may relate more to the relatively separate, sequence-general component of SISL Speed.

Personality constructs, specifically relating to openness, have also been studied in relation to implicit learning, though findings have been mixed. Kaufman et al. (2010) found SRT implicit learning to correlate with self-reported personality measures relating to the openness scale of the Big Five Aspect Scales (BFASs). Similarly, Sobkow et al. (2018) found that both SRT and AGL performance weakly related to specific aspects of the openness-to-experience personality domain. In contrast, openness, as measured by the Revised NEO Personality Inventory (NEO-PI-R), did not relate to sequence learning on a deterministic SRT task (Norman et al. 2006) or probabilistic SRT task (Norman et al. 2007). Here, these two extensively validated scales related to openness, BFAS and NEO-PI-R, were selected.

### 4.1. Participants

In Experiment 4, *n* = 220 participants were recruited through Northwestern’s Paid Participant Registry. Participants were compensated $10/h per session of the four-session study. Of 220, 55 (25%) were excluded in total, leaving *n* = 165 for analysis. Of the 55 excluded, 21 participants were excluded for having missing data from more than one session, and 34 participants were excluded for poor performance, with 22 of 34 having excessive missed responses, 9 having excess overall responses, and 3 having excessively low performance (<25% overall accuracy).

### 4.2. Materials

*SISL.* Participants completed the SISL task as described in Experiment 3 twelve times over three sessions. As in Experiment 3, participants completed four training and test block pairs (unique repeating sequences) in each session, with blocks of 60-trials of non-repeating foil segments (5 SOC sequences concatenated) used during each test block. New training and foil sequences were used for each training and test block pair. SSPA was calculated as the difference in accuracy between the repeating sequence blocks and these non-repeating blocks. In addition, Speed was reset back to the original level of 1.5 s (7.86 cm/s) to target at the beginning of each new training and test block pair to assess individual differences in speed performance more accurately. However, missed trials were not counted as errors due to a coding error, resulting in errors within the speed adjustment algorithm. Speed adjustments that should have normally occurred every 12 trials were not always correctly triggered, thus leading to lower performance than usual. In addition to SISL, participants were also given a battery of assessments measuring cognitive functions related to working memory, fluid intelligence, processing speed, and personality. Each task within the cognitive battery is detailed below.

*Operation Span.* Participants were instructed to solve a series of mathematical operations while simultaneously memorizing a set of unrelated words (Turner and Engle 1989). Mathematical operations were structured as a simple multiplication or division problem, such as (3 × 4) or (8/2), followed by the addition or subtraction of a single-digit integer (e.g., “Is (9/3) − 1 = 1?”). Words paired with the mathematical operation were monosyllabic concrete nouns that were four to six letters long. A typical trial consisted of evaluating the truth of the math statement, indicating whether it was correct, and subsequently viewing a word (e.g., “BALL”). Participants were instructed to read the word before moving on to the next operation–word pairing. At intervals of two to six trials, participants were asked to recall the words that accompanied the previous set of trials in the correct order, beginning at a set of two trials and moving sequentially up to a set size of six. Working memory capacity was scored as the sum of the number of words from each correctly recalled trial order, with a maximum score of 60.

*List Sorting.* The List Sorting Working Memory Test was taken from the National Institute of Health Toolbox Cognitive Function Battery (NIHTB) intended for online or computer-based use (Tulsky et al. 2013). Pictures of animals were presented for two seconds each, and participants were asked to recall the animals in the correct order for set sizes of two to seven animals. Sets progressed serially from two to seven, with two trial orders at each set size. Working memory span was scored as the set size at which participants correctly recalled at least one of the two trials orders.

*Sequential Visuospatial (SeVi) task.* This task is a novel measure that was intended to measure working memory span and was designed to look similar to the SISL task. As in SISL, participants viewed circular cues scrolling down a screen towards target circles labeled D, F, J, or K. Unlike SISL, instead of immediately responding, participants were instructed to remember the order of the cue sequence and repeat the order back after a delay. Cues were both red and blue in color, and participants were instructed to ignore the blue cues but remember the sequence of red cues. After all the cues disappeared from the screen and a ~1 s delay, participants attempted to repeat back the order of the red cue sequence. Participants were shown an initial sequence length of two items, which increased in a staircase fashion after two successive correct responses. Two consecutive incorrect responses led to a reduction in sequence length. Participants completed 30 trials of this task, which lasted approximately 5–10 min. Working memory span for cue sequences was scored as the longest sequence length at which the participant achieved an overall accuracy of 70% correct.

*Matrix reasoning.* The Matrix Reasoning test was used and was sourced from International Cognitive Ability Resource (ICAR), which was a set of public-domain assessment tools for various cognitive measures easily obtained for online use (Condon and Revelle 2014; Dworak et al. 2021). Matrix reasoning items of the ICAR included 3 × 3 arrays of geometric shapes with one of the nine shapes missing, similar to the Raven’s Progressive Matrices, which was commonly used to measure fluid intelligence (Raven et al. 1977). Eleven of these items were presented to participants. On each trial, participants were asked to decide which of the six possible shapes presented below the array best completed the pattern.

*Letter and number series.* The letter and number series items from ICAR was used (Condon and Revelle 2014), similar to the Thurstone (1940) letter series and number series tasks relating to inductive reasoning and fluid intelligence. This test consisted of nine items, and each item was a short letter or digit sequence. On each trial, participants were shown the short letter or digit sequence and were then prompted to identify the next position in the sequence from among six possible choices.

*Speed of information processing.* The Speed of Information Processing (SOIP) sub-test from the British Ability Scales was used (Elliott et al. 1996). Participants were shown sets of five integers, each randomly chosen from 1 to 100. Participants were then asked to select the highest number in each set and were given 60 s to complete as many of the 48 items as they could. The score for this task was the number of items completed correctly after 60 s.

*Digit-symbol substitution.* The Digit-Symbol Substitution task from the Wechsler Adult Intelligence Scale-Revised was used (Wechsler 1981), which was also commonly used in the aging cognition literature as an indicator of age-related cognitive decline in processing speed (Hoyer et al. 2004). A key of symbols was presented that matched with the number 1–9. Beneath the key were a series of symbols that were each followed by a blank box. For online purposes, participants were instructed to fill in the correct number that corresponded to that symbol in the key.

*Personality measures.* Participants completed two personality scales to measure openness to experience: the openness subscales of the Revised NEO Personality Inventory (NEO-PI-R) (Costa and McCrae 1992) and the Big Five Aspect Scales (BFAS) (DeYoung et al. 2014). Both scales asked participants to indicate the extent to which they agreed or disagreed with a series of descriptive statements (e.g., “I love to reflect on things”) on a 5-point scale. Individual items were scored from 1 to 5 based on participants’ responses, with items reverse-coded accordingly, and then summed across items, yielding a single “openness” score for each scale (NEO-PI-R max score = 170, BFAS max score = 50).

### 4.3. Procedure

Participants completed four one-hour sessions online. Following the completion of a session, the link to the next session was emailed to participants approximately 18–24 h later. In the first session, participants completed the two working memory tasks, the two personality measures, the two fluid intelligence measures, and the two information processing speed measures. These tasks were completed online via Qualtrics survey software. For the subsequent three sessions, participants completed SISL training and the SeVi task. Each session of the SISL task consisted of eight blocks of training and testing, with one block of each for the four different sequences identical to Experiment 3. Participants were given new sequences for each assessment per session (twelve unique sequences). Each session of the SeVi task was 30 trials long, as described in Section 4.2.

### 4.4. Results

**Figure 4:**
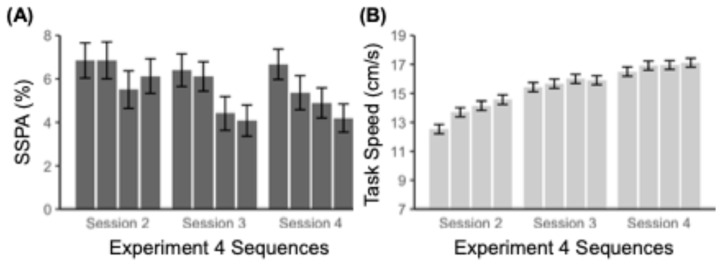
(**A**) Experiment 4 (*n* = 165) Sequence-Specific Performance Advantage (SSPA) means across 12 independent sequence learning assessments over three days (sessions 2–4). For all measures, brackets reflect the standard error of the means. Overall linear trends towards changes in SSPA across sequences indicated a significant decrease in sequence-specific performance across the 12 learned sequences. (**B**) Experiment 4 Speed improved consistently across sequences and sessions (days) following a log-linear structure.

Participants reliably learned all twelve sequences across Sessions 2–4 (Session 2: *M* = 6.30%, *SE* = 0.41%; Session 3: *M* = 5.24%, *SE* = 0.37%; Session 4: *M* = 5.28%, *SE* = 0.35%), *ts* > 5.62, *ps* < .001. The linear trend for the repeated measures ANOVA across the 12 sequences was significant, indicating a downward trend in SSPA across sequences, *F*(1, 160) = 8.46, *p* < .01, *η*^2^ = 0.050 (Figure 4A). A further breakdown by one-way repeated measures ANOVAs of SSPA across the four unique sequence tests within each session indicated that the significant linear trend occurred for Session 3 (*F*(1, 162) = 6.09, *p* = .014, *η*^2^ = 0.036, *BF* = 3.04) and Session 4 (*F*(1, 163) = 6.40, *p* = .012, *η*^2^ = 0.038, *BF* = 0.08) but not Session 2 (*F*(1, 163) = 0.79, *p* = .37, *η*^2^ = 0.0048, *BF* = 1.46). While foil learning was controlled for in this iteration, the downward trend of SSPA in later sessions here hints at potential learning occurring at the trigram level, though is more likely an artifact of measurement noise. See Table A2 for further information.

A linear trend for the one-way repeated measures ANOVA of Speed was significant across the four sequence tests for Session 2 (*F*(1, 163) = 134.35, *p* < .001, *η*^2^ = 0.45), Session 3 (*F*(1, 162) = 17.10, *p* < .001, *η*^2^ = 0.10), and Session 4 (*F*(1, 163) = 19.11, *p* < .001, *η*^2^ = 0.10). The linear trend for the repeated measures ANOVA was also significant across all 12 sequences (Session 2: *M* = 13.75 cm/s, *SE* = 0.16 cm/s; Session 3: *M* = 15.71 cm/s, *SE* = 0.16 cm/s; Session 4: *M* = 16.88 cm/s, *SE* = 0.16 cm/s), *F*(1, 160) = 541.97, *p* < .001, *η*^2^ = 0.77. This suggests that participants generally improved at the task across sessions (Figure 4B).

**Figure 5:**
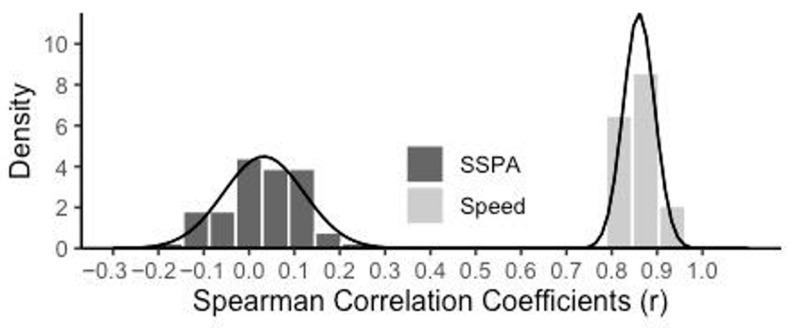
Density plots of Spearman correlation coefficients for Sequence-Specific Performance Advantage (SSPA; dark gray) and Speed measures (light gray) across all 12 sequence assessments in sessions 2–4 (66 possible pairwise comparisons) with superimposed normal distribution curves. High correlation values indicate rank-order stability across measures. Participants who were faster at the task were consistently faster on all assessments, leading to high correlation coefficients (mean *ρ* = 0.86). In contrast, sequence learning scores did not correlate well across sequences (mean *ρ* = 0.03), indicating that, in general, no participants were observed to be consistently better or worse at sequence learning.

As in Experiments 1–3, individual differences in SSPA were low but higher than the previous experiments (absolute agreement ICC = 0.30, consistency ICC = 0.31). In contrast to SSPA, like previous experiments, Speed was highly reliable and consistent (absolute agreement ICC = 0.98, consistency ICC = 0.99). The inclusion of 12 parallel-form measures of SISL performance allows for a novel approach to examining the range of cross-assessment correlation in performance for SSPA and Speed measures. All 66 possible pairwise correlations (between each pair of sequences) for both SSPA and Speed were calculated and evaluated as a distribution (Figure 5). The Spearman correlation coefficients for SSPA were near zero (average *ρ* = 0.033), again suggesting that even across 12 independent assessments over 3 days, no evidence was observed for some participants being better sequence learners. In striking contrast, the Speed measures of task general performance were highly consistent, with very strong correlations across all measures on all days (average *ρ* = 0.86).

#### 4.4.1. Correlations with the Cognitive Battery

**Figure 6:**
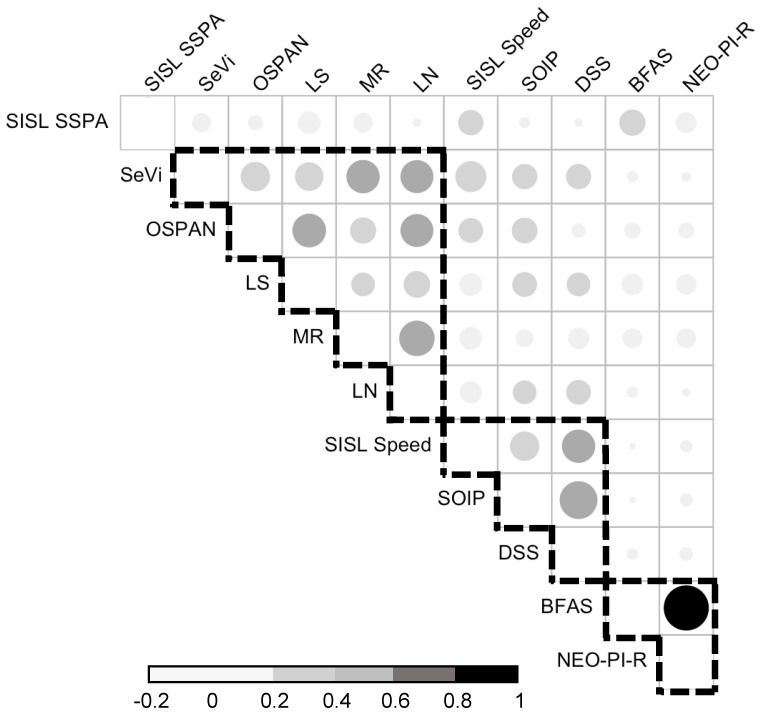
Correlation matrix among all measures, including mean of 12 sequence learning measures (SISL SSPA), working memory and fluid intelligence measures (LS = List Sorting, OSPAN = Operation Span, SeVi = Sequential Visuospatial, MR = Matrix Reasoning, LN = Letter and Number Series), the average of 12 SISL Speed measures, processing speed measures (SOIP = Speed of Information Processing, DSS = Digit Symbol Substitution), and personality measures (Openness on NEO-PI-R and BFAS = Big Five Aspect Scales). The size of the circles indicates the absolute value of the correlation coefficient, and color indicates the direction of the correlation.

Figure 6 shows correlations among all cognitive measures completed during the first session, with SISL SSPA, SISL Speed, and SeVi working memory span averaged across sessions 2–4, and with *p*-values corrected for multiple tests. See Table A3 for descriptive statistics of the cognitive battery. Assessments that have been previously reported to measure the same construct tended to have the highest correlations within the battery: Operation Span and List Sorting (working memory): *r* = 0.44, *p* < .001; Matrix Reasoning and Letter Number Series (fluid intelligence): *r* = 0.49, *p* < .01; Speed of Information Processing and Digit Symbol Substitution (processing speed) tasks: *r* = 0.56, *p* < .001; NEO-PI-R and BFAS scales (openness personality aspect): *r* = .81, *p* < .001. Additionally, there were moderate correlations between most of the different cognitive measures, particularly the working memory and fluid intelligence measures, *rs* [0.21, 0.49], *ps* < .01.

The average SeVi score was also significantly correlated with the other working memory measures, *rs* [0.31, 0.33], *ps* < .001, and fluid intelligence measures, *rs* [0.42, 0.43], *ps* < .001. The SISL Speed measure was also significantly correlated with other processing speed measures, *rs* [0.33, 0.43], *ps* < .001. In contrast, and in line with the previous experiments, SSPA had low and non-significant correlations with the other measures, *rs* [0.02, 0.19], *ps* > .05, except for an openness personality measure (BFAS), *r* = 0.26, *p* < .05, which was likely spurious, and average SISL Speed, *r* = 0.24, *p* < .05. Additionally, the average Speed and SeVi scores were correlated, *r* = 0.37, *p* < .001, whereas the average SSPA score did not correlate with the average SeVi score, *r* = 0.13, *p* = .09.

The inclusion of 12 parallel-form measures of SISL performance allows for a novel approach to examining the range of cross-assessment correlation in performance for SSPA and Speed measures. All 66 possible pairwise correlations (between each pair of sequences) for both SSPA and Speed were calculated and evaluated as a distribution (Figure 5). The Spearman correlation coefficients for SSPA were near zero (average *ρ* = 0.033), again suggesting that even across 12 independent assessments over 3 days, no evidence was observed for some participants being better sequence learners. In striking contrast, the Speed measures of task general performance were highly consistent, with very strong correlations across all measures on all days (average *ρ* = 0.86).

#### 4.4.2. Factor Analysis with Cognitive Battery

**Figure 7:**
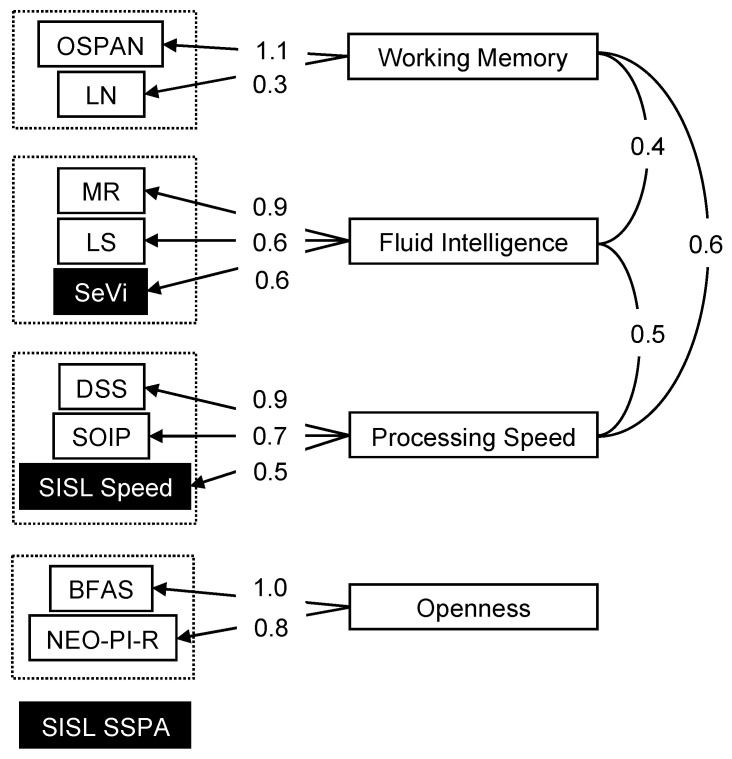
Strength of factor analysis loadings for all Experiment 4 cognitive measures (Promax rotation). Solid arrows indicate strong factor loadings to a stable factor. Measures include the average of 12 sequence learning measures (SISL SSPA), 2 working memory measures (OSPAN = Operation Span, LS = List Sorting), 3 fluid intelligence measures (MR = Matrix Reasoning, LN = Letter and Number Series, SeVi = Serial Visuospatial), the average of 12 SISL Speed measures (SISL Speed), 2 processing speed measures (SOIP = Speed of Information Processing, DSS = Digit Symbol Substitution), and 2 personality measures (Openness on NEO-PI-R and BFAS). Tests that assessed working memory (WM), fluid intelligence (gF), processing speed, and personality (Openness) each loaded onto a consistent factor. Correlations between the working memory, fluid intelligence, and processing speed factors were found. The SeVi measure, which was designed to appear like the SISL task, loaded with the fluid intelligence factor. The Speed measure obtained during SISL performance loaded onto the Processing Speed factor, but the sequence learning measure (SSPA) did not strongly load onto any of the stable underlying factors identified.

Following correlation analyses, a factor analysis was used to further explore the relationship between the cognitive and implicit learning measures, using averaged SSPA, Speed, and SeVi scores from the three sessions (Figure 7). A three-factor solution was supported by the parallel method of factor dimension extraction, which compares the scree of successive eigenvalues in observed data with that of a random data matrix the same size as the original (Revelle 2017). The three-factor solution was analyzed with a Promax rotation, which is an oblique rotation method that assumes the factors can be correlated.

Statistical criteria for good fit included a Root Mean Square Error of Approximation (RMSEA) of <0.06 for a close fit and <0.08 for a reasonable fit (Browne and Cudeck 1993; Hu and Bentler 1999), Standardized Root Mean Residual (SRMR) < 0.05 for a good fit (Byrne and Russon 1998; Hooper et al. 2008) and value < 0.08 for a reasonable fit (Hu and Bentler 1999); and Comparative Fit Index (CFI) and Tucker–Lewis Index (TLI) > 0.95 for a good fit (Hu and Bentler 1999; Hooper et al. 2008), and values < 0.90 to 0.95 show a reasonable fit (Kline 2023). The three-factor model fit adequately by some criteria but not by others (CFI = 0.94, TLI = 0.87, RMSEA = 0.08, SRMR = 0.05). Thus, a four-factor model was analyzed with a Promax rotation, and this solution provided an improved fit that was relatively close-fitting by most criteria, indicated by CFI = 0.98, TLI = 0.93, RMSEA = 0.07, SRMR = 0.04.

The working memory measures and the fluid intelligence measures loaded onto separate factors and were also correlated at 0.4, consistent with prior work that indicated working memory is strongly related to fluid intelligence (Conway et al. 2003). SISL Speed and the information processing tasks loaded onto a second factor, which we describe as a processing speed factor, with a between-factor correlation of 0.5 with the fluid intelligence and 0.6 with working memory. The medium between-factor correlation between working memory and processing speed hints at an underlying higher order latent construct underlying these two factors, which is consistent with work in psychometric intelligence. The two personality measures loaded most strongly onto a fourth factor, which we describe as an openness factor, while SISL SSPA did not load strongly onto any factor.

In addition to the significant correlation between the average SSPA and Speed in the cognitive battery, the moderate and significant correlations between SSPA and Speed in the previous experiments as well as non-zero factor loading of SSPA onto processing speed invites a more precise investigation of the nature of this relationship. While findings thus far have confirmed the relative instability of sequence-specific learning across unique sequences, Speed seems to be a more reliable measure of individual differences. The correlation between SSPA and Speed, while low, suggests that the relationship between SSPA and Speed hints at the possibility of a weak individual differences signal in SSPA.

### 4.5. Experiment 4 Discussion

Experiment 4 further examined the question of whether we could measure stable individual differences in implicit sequence learning. Here, we used a substantially larger sample of participants (*n* = 165) and twelve unique parallel-form sequence learning measures over three days. Once again, no evidence was found to support the idea that some people were substantially better than others at implicit sequence-specific knowledge, as observed in the previous Experiments 1–3. Though, ICCs were found to be higher in this experiment compared with Experiments 1–3, possibly due to the increased number of sequence assessments. Despite this, the obtained value is still qualitatively considered “poor”. In addition, we employed a cognitive battery measuring working memory capacity, fluid intelligence, processing speed, and personality (openness) constructs to identify whether these cognitive measures strongly correlated with the sequence-specific learning measure (SSPA). Yet, these instruments produced a typical pattern of inter-test correlation as found in other psychometric work, with the exception of a weak but reliable correlation between SSPA and the openness personality construct, as measured by the Big Five Aspect Scales openness subscale.

Although the sequence-specific learning measures did not exhibit reliable differences across parallel form measurements, the sequence-general Speed measure that was simultaneously measured alongside sequence-specific SSPA continued to exhibit robust stability across tests. As might be expected, this measure also substantially loaded onto other cognitive measures related to processing speed. The stability of the Speed measure and its relationship to standard psychometric measures suggests that the lack of stability in SSPA is not due to some aspect of the SISL methodology that produces noisy or high variance measures in general. Across all SISL assessments, we consistently observed reliable sequence learning (SSPAs > 0), but there was no evidence that higher scores on one assessment predicted better learning on other assessments.

We also introduced a novel Sequential Visuospatial (SeVi) task in this study, which was intended to provide a measure of working memory capacity using an interface designed to match the SISL task. Interestingly, this measure of sequential visuospatial span was correlated with other standard measures of working memory capacity as well as fluid intelligence and loaded onto the fluid intelligence factor. The expected relationship of both the SISL Speed and SeVi measures to other well-studied cognitive measures suggests that the lack of stable differences in implicit learning as measured by SSPA is not due to some methodological aspect of our task, interface, or general data collection approach.

While the absence of evidence for trait differences in implicit learning cannot unambiguously establish that there are absolutely no differences in implicit learning across people, the data at least argue for a very small effect size. At a minimum, differences between individuals in sequence learning look to be substantially smaller than differences found in other cognitive and personality constructs, which were found to be robust here. Experiment 4 reinforces the observations from Experiments 1–3 that sequence-specific knowledge does not appear to be an individual trait, even when assessed in parallel with a range of measures that exhibit typical trait-like consistency, such as working memory capacity, processing speed, and personality.

## 5. General Discussion

Across four experiments, participants completed two, four, or twelve parallel forms of SISL sequence learning assessments with a unique novel repeating sequence embedded in each assessment. Each assessment remeasured the learning rate for new sequential information within the SISL implicit learning task with novel unfamiliar material. Participants exhibited robust sequence-specific learning for each form in each experiment. Across all four experiments, however, there was no evidence for consistent interindividual differences in implicit learning of sequential information. In each experiment, participants learned on each repeated assessment, but no participants exhibited consistently better learning, as would be expected by the existence of a better overall learning ability as an individual trait. We consistently observed effective learning, but we did not find strong evidence for a trait-like ability for implicit learning that would lead some individuals to consistently learn at a faster (or slower) rate than others.

Although the lack of rank-order stability across assessments is fundamentally a null result, it should be noted that the repeatedly reliable learning across every form used with SISL indicates that the lack of inter-assessment correlation cannot be due to floor effects. In addition, the implicit learning measure SSPA has been previously shown to continue to increase log-linearly with practice (Sanchez and Reber 2012) such that the learning gains here reflect relatively lower amounts of practice and clearly not ceiling effects. Of course, we cannot rule out the possibility that individual differences in learning rate are simply very small, especially since our learning assessments were all quite short to allow for repetition across the parallel forms. Even a small difference in learning rate could lead to more robust individual performance differences across thousands of hours of practice. Our results are consistent across four experiments using up to twelve repeated reassessments of learning rate that everybody learns, but at this scale, no one consistently learned better than anyone else.

An additional finding about sequence learning within SISL embedded within our parallel-forms approach is that in no experiments did we observe any interference among subsequent learning processes that started over with a novel repeating sequence. Nor did we observe any learning-to-learn effects, whereby with practice, participants became better at extracting the statistical structure of the repeating sequence to more effectively guide responses. Both of these observations would be very surprising for learning material learned consciously. Even for an implicit learning task, the lack of any effect of learning a prior sequence on the next sequence potentially illustrates aspects of the underlying learning mechanisms. Simple statistical models of learning transitional probabilities or associations might predict effects based on partial overlap of randomly selected sequences (and the foils used to demonstrate learning), but the absence of these effects suggests a more complex statistical learning mechanism is involved.

Although the sequence-specific learning did not show stable individual differences, the individually adaptive Speed measure showed very robust and consistent differences in each of the four experiments. Participants who were faster were consistently faster and in Experiment 4, this task speed measure correlated strongly with standard measure of processing speed. The rank-order correlations of the Speed assessments across reassessments of learning were consistently at the .90 or greater level across experiments, reflecting very high levels of individual consistency. Notably, Speed as measured by the SISL task is not pure reaction time to the presentation of a cue, in contrast to common implicit learning tasks like SRT, but rather the speed at which precisely timed motor responses can be made at a particular cue velocity. Because the adaptive speed algorithm dynamically adjusts the cue velocity at the sequence level, regardless of whether the sequence is trained or untrained, Speed reflects sequence-general performance on the task. When we created a working memory assessment within the same task user interface (SeVi), performance on this measure correlated strongly with other standard working memory and fluid intelligence measures. These observations serve to show that the lack of individual sequence-specific learning on the SSPA measure cannot be attributed to some peculiarity of the task presentation, design, or online implementation.

Our results stand somewhat in contrast to prior studies that had reported some evidence of correlated individual differences across implicit learning tasks, including some weaker evidence of relationships with other cognitive measures (e.g., Kaufman et al. 2010; Kalra et al. 2019). We hypothesize that the vulnerability to explicit knowledge of many implicit learning protocols may have allowed the scores to be influenced by cognitive processes known to have stable individual differences. For example, contamination of the learning scores by conscious cognition via working memory (Janacsek and Nemeth 2013) could potentially cause correlations among scores due to individual differences in working memory rather than implicit learning rate. The relatively unique resistance of the SISL task to increased performance scores based on explicit knowledge (Sanchez and Reber 2013) and the ability to separate out the role of processing speed from learning performance provided here a unique opportunity to directly assess any individual differences in implicit learning. It likely requires a relatively more process-pure task like SISL to isolate the implicit learning process from other cognitive functions to assess operating characteristics of this form of learning.

### 5.1. Limitations

We acknowledge that our results may be affected in a manner similar to the ongoing discussion regarding the “reliability paradox” across various cognitive tasks (Hedge et al. 2018), including SRT (Oliveira et al. 2023), which refers to low psychometric reliability and stability of test scores across sessions despite robust group-level learning. Sequence-specific knowledge, as measured through the SSPA, likely includes some variance due to measurement error, as every cognitive measure does. However, we demonstrated moderate test–retest reliability for the same statistical information via a split-half correlation in Experiment 2, showing that the measures do effectively estimate the amount learned. This analysis, used in some other studies of implicit learning, addresses how well the learning measure reflects the underlying construct of amount learned. The parallel forms methodology used here provides a relatively unique approach to measuring reliability of learning rate by reassessing learning rate repeatedly, not just testing for test–retest reliability of a single measure.

We also acknowledge that there are methodological limitations that should be addressed. The set of studies here did not include direct measures of explicit sequence knowledge. Although we cannot definitively rule out that some participants acquired explicit knowledge of the sequence, previous work (Sanchez et al. 2010) supports the view that performance improvements on the SISL task can emerge independently of awareness. In particular, when participants were explicitly informed of the repeating sequence before training, their sequence-specific performance did not differ from that of uninformed, naïve participants (Sanchez and Reber 2013). Nevertheless, we recognize that this assumption may not generalize across all implementations and should be verified directly in future work.

Additionally, the continued adjustment of speed during the test phase represents a design choice with both strengths and limitations. On one hand, adjusting the cue velocity throughout testing ensures that participants remain engaged at the appropriate difficulty level (set to 80% overall). On the other hand, this adaptive mechanism may potentially reduce sensitivity to sequence-specific performance by elevating the performance accuracy of both trained and untrained sequences, especially during the test phase which contains a higher proportion of untrained sequences. While this may lead to more conservative estimates of SSPA, the observed effects remain robust, as SSPA emerged at the group-level consistently across all experiments described here despite these conditions. Future work could examine alternate implementations, such as holding the cue velocity constant during the test phase. Future work could also investigate whether different task difficulty levels corresponding to higher (e.g., 90%) or lower (70%) accuracy targets influence the magnitude of SSPA. These variations may help determine whether SSPA is more sensitively detected under conditions that are tuned to elicit different ranges or levels of performance across individuals.

Our results also do not rule out the possibility that some participants are substantially worse at skill learning. Our methodology typically excludes participants whose performance is particularly poor (19–25% of participants across experiments), and, while we generally expect that this exclusion criteria applies to participants who are non-compliant with task instructions, this may also accidentally exclude very poor learners. Our data were also collected through online sources with participants who voluntarily chose to be in the study, which might also create selection bias away from participants who chose not to participate in these research studies for reasons related to feeling like they find rhythm games challenging.

### 5.2. Future Research

The reliance on a single task in the studies presented here does raise a question of whether the results here are specific to perceptual-motor sequence learning or even the specific cognitive processes required to perform and learn during SISL tasks. We hypothesize that the term implicit learning refers to a broad capacity for neural reorganization from repeated practice (Reber 2013) but acknowledge the possibility that there may be a collection of distinct mechanisms that contribute to this form of learning. Different neuroplasticity mechanisms that produce learning from cortical–cortical connections, basal ganglia, or cerebellar loops may produce different patterns of operating characteristics or individual differences across paradigms. To identify these, it will be necessary to develop additional process-pure learning paradigms that allow those learning processes to be isolated from other, related cognitive functions. In the experiment here, we show that for implicit perceptual-motor sequence learning, isolating this cognitive process identifies a learning mechanism that is present in all participants but does not vary substantially across them.

Additionally, while we relied on accuracy-based metrics to estimate sequence-specific learning, temporal precision represents a potentially informative and complementary measure of individual differences. Here, a response is scored as correct if the cue falls within a predefined response window surrounding the target location. This approach inherently reflects a form of temporal precision, but our outcome variable reduces this information to binary correct or incorrect responses. While SSPA has been widely used in a number of prior SISL studies (Sanchez et al. 2010; Gobel et al. 2011; Sanchez and Reber 2012, 2013; Thompson et al. 2014; Damme et al. 2023), it is possible our current approach lacked the sensitivity to detect subtle individual differences in sequence-specific motor timing. Individual differences related to motor timing, such as sensorimotor synchronization in simpler tapping tasks, has been shown to vary systematically across individuals (Lorås et al. 2013), particularly musicians compared with non-musicians (Repp 2010). While these findings point towards greater variability with relation to processing speed rather than sequence-specific learning, it is possible subtle variance in sequence-specific precision may have been missed, and we acknowledge this is a valuable dimension of performance. We have begun work in separate studies examining temporal precision of motor responses using SISL in both neurotypical and clinical populations (e.g., individuals at high risk for psychosis; Damme et al. 2023). Future research incorporating metrics of temporal precision may provide additional insight into implicit learning-related individual differences.

The idea that implicit learning as an ability does not vary substantially across the population may have implications for the study of expertise, since expertise is often acquired during a process of very large numbers of repetitions. No differences could be seen as arguing against the idea that some individuals have greater innate talent leading them to higher levels of extraordinary expert performance. Campitelli and Gobet (2011) have argued that standard models of expertise acquisition are insufficient to account for the observed differences among experts at the very highest level of performance. Ericsson (2004) proposed that differences among experts were best characterized as emerging from differences in *deliberate practice*, which reflected how instruction, coaching, and guidance during repeated practice would lead to different levels of expert accomplishment (Ericsson et al. 1993; Ericsson and Charness 1994). Our results are consistent with a memory system’s interpretation of deliberate practice reflecting a combination of explicit, conscious instruction directing a repetition-based implicit learning process (e.g., by practicing the most effective action sequences). However, using chess expertise as an example, Campitelli and Gobet (2011) argue that among the very best experts in the world, performance varies too much to be accounted for by the quality of training, and the very best performers in the world appear to exhibit a talent advantage. A challenge embedded in resolving this question is the potential that an innate talent advantage exists but is very rare. Studies like the ones reported here would not be powerful enough to detect a single unusual individual who does actually learn at a faster rate. It is likely necessary to study selected extraordinary performers to address this question or to do extremely large-scale population studies capable of identifying a small number of unusually effective learners.

## 6. Conclusions

Using a unique method of multiple parallel-forms reassessments of learning rate, made possible by a unique task that allows for a process-pure measure of implicit learning, we find no real evidence for stable individual differences in ability. Everyone exhibited implicit learning, but no participant was observed to be consistently better at learning than others. This similarity reflects our sampling methodology and may not have included rare cases of unusual talent (or unusual challenge) but is consistent across four experiments with robust numbers of participants. The lack of consistent differences cannot be due to floor or ceiling effects, nor can it be due to measurement error due to the particular implementation of our task. With the same implementation, we observed robust individual differences in processing speed and working memory capacity, just not in implicit learning rate. Explicit cognitive processes like working memory capacity vary substantially and consistently across people. In contrast, we hypothesize that implicit learning arises from neural plasticity functions that appear to operate to produce similar learning rates across people. Our results may reflect a consistency specific to perceptual-motor sequence learning, but the task used here may reflect a general characteristic of the hypothesized process of naturally occurring slow reorganization of neural function that improves efficiency and accuracy through statistical abstraction during repetition.

## Figures and Tables

**Figure A1:**
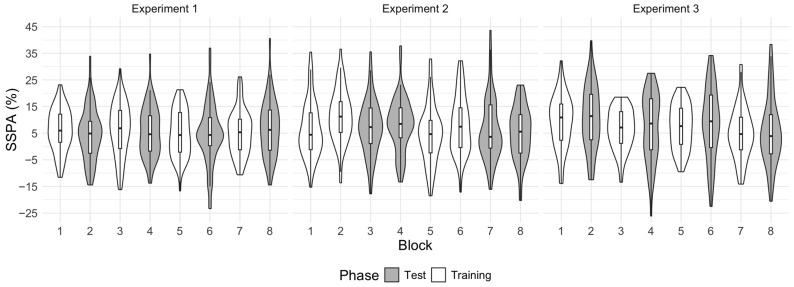
Sequence-Specific Performance Advantage (SSPA) for each training and test block across Experiments 1–3. Note that each training and test block are 540 trials in length.

**Figure A2:**
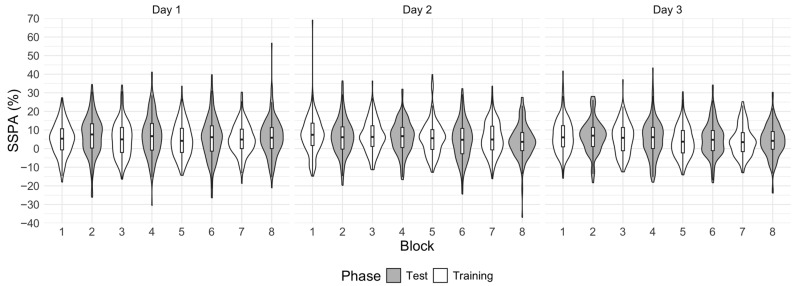
Violin and overlaid box plots of the Sequence-Specific Performance Advantage (SSPA) for each training and test block across the multiple days of Experiment 4. Note that each training and test block are 540 trials in length.

## Data Availability

Data supporting the results can be found in OSF at https://osf.io/n3y2w/, accessed on 27 June 2025.

## Abbreviations

The following abbreviations are used in this manuscript:

AGL	Artificial Grammar Learning
ASRT	Alternating Serial Reaction Time
ICC	Intraclass Correlation
IQ	Intelligence Quotient
SeVi	Sequential Visuospatial
SISL	Serial Interception Sequence Learning
SOC	Second Order Conditional
SRT	Serial Reaction Time
SSPA	Sequence-Specific Performance Advantage
VMA	Visuomotor Adaptation

## Appendix A. Experiment 2

To include four parallel form assessments of sequence learning, each assessment in Experiment 1 was shorter than prior SISL studies (36 sequence repetitions instead of ~200). Although reliable learning was observed on all sequences, this shorter training period may not have provided enough time for individual differences in sequence-specific knowledge to emerge. For Experiment 2, we replicated the multiple assessment approach but with just two parallel form assessments to allow for longer training and test phases for each of the sequences learned. In addition to the repeated assessment of sequence learning, the longer test phase allowed for a direct measure of test–retest reliability for SISL within the same sequence.

### Appendix A.1. Participants

In Experiment 2, *n* = 74 participants were recruited through Amazon’s Mechanical Turk online interface (demographics therefore unavailable), excluding participants who had been in Experiment 1. A total of 14 participants were excluded for non-compliance (20%), leaving 60 participants for analyses. Of the 14 excluded, 2 had excessive missed responses, 6 excess responses, and 6 had excessively low performance (<25% overall accuracy) in at least one 180-trial sub-block.

### Appendix A.2. Procedure

Two parallel form assessments of sequence learning using the SISL task were completed by each participant following the same basic procedure as Experiment 1. For Experiment 2, the training phase consisted of two 540-trial blocks to provide greater learning opportunity. In addition, the test phase was also doubled in length to 1080 trials (6 60-trial subblocks of repetitions of the repeating sequence and 12 60-trial subblocks of two repeating foils). As in Experiment 1, the starting speed for the second session was the same as the final speed obtained at the end of the first test block.

### Appendix A.3. Results

Participants reliably learned both sequences and the average group SSPA measure for the first sequence test (*M* = 7.69%, *SE* = 1.10%, *t*(59) = 7.0, 95% CI [5.49%, 9.88%], *p* < .001, Cohen’s *d* = 0.91) and the second sequence test (*M* = 5.79%, *SE* = 1.09%, *t*(59) = 5.34, *p* < .001, 95% CI [3.62%, 7.97%], Cohen’s *d* = 0.69) did not reliably differ, *t*(59) = 1.21, *p* = .23, *95% CI* [−1.24%, 5.03%], Cohen’s *d* = 0.16, *BF* = 0.28 (Figure 3A). The Spearman correlation between the two SSPA measures was not reliable and approximately zero, *ρ*(58) = −0.15, *p* = .26. Like Experiment 1, individual differences in SSPA were low (absolute agreement ICC = 0, consistency ICC = 0).

To verify that the SSPA measure reliably assesses sequence knowledge, internal consistency via Pearson split-half correlations across the two test blocks were examined. During the first sequence test, participants’ SSPA across blocks were reliably correlated, *r*(58) = 0.42, *p* < .001, *BF* = 46.6. However, the split-half correlation for the second sequence was only marginal, *r*(58) = 0.23, *p* = .07, *BF* = 1.29. These results indicate that the SSPA measure of sequence learning has measurement error embedded, which may be contributing to the lack of observed correlations between assessments. However, within-sequence correlations were found to be reliably higher than the between sequence correlation, z = 3.47, *p* < .001 (Fischer r to z transformation), indicating that the SSPA score is much more strongly dependent on sequence knowledge than individual differences in sequence-specific knowledge.

The Speed measure reliably improved across assessments (from *M* = 12.31 cm/s, *SE* = 0.48 cm/s to *M* = 13.20 cm/s, *SE* = 0.49 cm/s), *t*(59) = 5.18, *p* < .001, *95% CI* [0.049 cm/s, 0.11 cm/s], as in Experiment 1 (Figure 3B). Speed was again highly correlated between the two sequence tests, *ρ* (58) = 0.93, *p* < .001. In Experiment 2, the correlation between Speed and SSPA (averaged across assessments) was reliable, *r*(58) = 0.28, *p* = .03, *BF* = 2.41. Individual differences and rank-order stability in Speed were, like in Experiment 1, substantial (absolute agreement ICC = 0.95, consistency ICC = 0.96).

### Appendix A.4. Experiment 2 Discussion

Experiment 2 replicated the findings of Experiment 1 with reliable sequence learning across multiple assessments but still did not provide evidence for stable trait-like differences in individual sequence-sequence learning. Within sequence assessment test–retest reliability in SSPA was moderate, raising questions about the sensitivity of our research protocol to individual differences in learning ability. However, the test–retest correlations were reliably larger than correlations across different assessments, suggesting that any trait-like ability in SISL sequence-specific learning is a relatively small effect. In contrast, the sequence-general ability reflected in the Speed measure was again highly stable across participants and clearly consistent with robust individual differences.

The highly stable Speed measure reflects an adaptive algorithm that adjusts the speed based on individual performance in order to maintain an overall accuracy level of 80% correct (so that SSPA can be calculated accurately). For Experiments 1 and 2, the adaptive speed settings were maintained across parallel assessments, which may have caused this measure to appear even more stable. For Experiment 3, we reset the speed algorithm at the beginning of each learning phase to determine if the measure obtained from a reconvergence of the algorithm continued to reflect stable individual differences.

For Experiment 3, we also considered the possibility that the standard method of measuring SSPA might inadvertently reduce individual differences. The SSPA measure is obtained during a test where participants perform SISL during blocks of their trained repeating sequence and blocks of untrained foil sequences. However, if participants are learning the foil sequences, the subtraction of these two accuracy measures could potentially remove some of the variance in performance associated with individual sequence-specific knowledge at test (faster sequence learners would also be faster foil learners). In Experiment 3, the test methodology was adjusted to contrast the trained sequence with long segments that contained no repeating structure to avoid the possibility that the foils were learned during the test.

## Appendix B. Experiment 3

### Appendix B.1. Participants

In Experiment 3, *n* = 65 Northwestern University undergraduate students participated for course credit, and 14 participants (22%) were excluded, leaving 51 participants for analysis. Of the 14 excluded, 2 had excessive missed responses, 7 had excess overall responses, and 5 had excessively low performance (<25% overall accuracy) in at least one 180-trial sub-block.

### Appendix B.2. Procedure

As in Experiment 1, participants completed four parallel forms SISL assessments (540 trials, 36 sequence repetitions during training). During the test blocks, instead of repeating foil sequences, the contrasting condition was constructed as blocks of 60-trials of non-repeating segments (5 SOC sequences concatenated to maintain balanced key frequency and first-order statistical structure). New training and foil sequences were used for each training and test block pair. SSPA was calculated as the difference in accuracy between the repeating sequence blocks and these non-repeating blocks. In addition, Speed was reset back to the original level of 1.5 s (7.86 cm/s) to target at the beginning of each new training and test block pair to assess individual differences more accurately in speed performance.

### Appendix B.3. Results

Participants again reliably learned all four sequences, *M* = 8.08%, *SE* = 0.89%, *ts* > 3.1, *ps* < .01 (Figure 3A). Once again, there was no evidence for order effects across assessments, *F*(1, 50) = 3.73, *p* = .059, *η*^2^ = 0.069, *BF* = 0.68, indicating no interference, fatigue, or learning-to-learn effects. Once again, Spearman correlations in SSPA among the four sequence tests were small and not significant (*ρs* [−0.035, 0.048], *ps* [.74, .99], *p*-values corrected for multiple tests). Similarly to Experiments 1–2, small ICCs indicate large heterogeneity among individuals. Individual differences in SSPA were not robust, with the calculated ICC being close to 0 (absolute agreement ICC = 0.01, consistency ICC = 0.01).

Also consistent with Experiments 1 and 2, participants’ Speed increased across assessments from *M* = 15.05 cm/s, *SE* = 0.54 cm/s to *M* = 17.16 cm/s, *SE* = 0.45 cm/s, *F*(1, 50) = 62.82, *p* < .001, *η*^2^ = 0.56, *BF* = 23.6 and were highly correlated, Spearman *ρs* range [0.72, 0.90], *ps* < 0.001 (Figure 3B). The Pearson correlation between average Speed and SSPA across the four sequence tests was significant, *r*(49) = 0.30, *p* = .04, *BF* = 2.36, with SSPA tending towards being slightly lower at higher speeds. Individual differences in Speed were, like in Experiment 1, “substantially” stable (absolute agreement ICC = 0.94, consistency ICC = 0.96).

### Appendix B.4. Experiment 3 Discussion

Changing the SSPA contrast condition to avoid repetition of previously unpracticed sequences led to slightly higher scores than Experiments 1–2 but provided no evidence of stable performance across assessments. In all experiments, robust sequence learning was consistently observed for multiple assessments within the experimental session (Figure 3A), but no evidence was observed for participants being generally better (or worse) at sequence-specific learning. The sequence-general Speed measure, on the other hand, produced highly consistent and stable differences between individuals (as well as learning across assessments; see Figure 3B). Correlations across Speed from multiple assessments were very robust in Experiments 1–3, indicating that participants who worked at higher speeds did so consistently across parallel SISL assessments. Absolute agreement and consistency ICC estimates consistently demonstrated that SSPA was not consistently stable across assessments, whereas Speed was both highly reliable and consistent.

Curiously, all three experiments also found some evidence of a relationship between participants’ Speed and SSPA measures, although SSPAs did not correlate with each other. This could indicate that the SSPA measure contains some smaller individual differences factors, or it could indicate some level of interdependence between the two measures. The link between processing speed and implicit learning has been suggested in prior work, though correlations were also generally low-to-medium in strength (Kaufman et al. 2010; Salthouse et al. 1999).

The lack of reliable stability across parallel forms of SISL could be due to Experiments 1–3 lacking the power to detect an effect that could be relatively small. In Experiment 4, we expanded our research protocol to a multi-day experimental session that included 12 parallel form SISL assessments of sequence learning completed across three days of testing. We also included a battery of common cognitive tests with known sensitivity to individual differences, including working memory capacity, processing speed, fluid intelligence, and personality measures. We also increased the number of participants in the study to increase the statistical power to detect subtle individual differences in performance.

## Appendix C

**Table A1:** Accuracy for trained and untrained sequences for each training and test block in Experiments 1–3.

Experiment	Block	Phase	*n*	Trained Sequence *M (SD)*	Untrained Sequence *M (SD)*	SSPA *M (SD)*
1	1	Training	60	63.57% (7.87%)	57.39% (8.87%)	6.18% (7.97%)
1	2	Test	60	60.82% (8.75%)	56.22% (9.23%)	4.6% (9.83%)
1	3	Training	60	58.85% (8.74%)	52.47% (12.5%)	6.38% (9.96%)
1	4	Test	60	59.99% (11.24%)	54.54% (9.70%)	5.44% (9.35%)
1	5	Training	60	59.70% (9.01%)	54.87% (11.61%)	4.83% (8.6%)
1	6	Test	60	61.70% (11.42%)	56.94% (10.76%)	4.76% (11.4%)
1	7	Training	60	58.75% (10.22%)	53.5% (11.71%)	5.25% (8.43%)
1	8	Test	60	62.64% (10.39%)	55.93% (10.48%)	6.71% (10.47%)
2	1	Training	60	62.66% (8.03%)	56.35% (8.29%)	6.31% (10.38%)
2	2	Training	60	61.40% (8.32%)	49.97% (11.23%)	11.43% (9.82%)
2	3	Test	60	63.15% (10.88%)	55.98% (8.13%)	7.18% (10.18%)
2	4	Test	60	63.44% (10.71%)	55.24% (8.4%)	8.2% (9.99%)
2	5	Training	60	59.88% (6.32%)	55.43% (11.18%)	4.45% (10.69%)
2	6	Training	60	62.05% (8.21%)	54% (11.85%)	8.06% (10.81%)
2	7	Test	60	64.25% (8.52%)	57.83% (9.51%)	6.41% (11.63%)
2	8	Test	60	63.56% (9.38%)	58.38% (7.98%)	5.18% (9.73%)
3	1	Training	51	69.20% (6.11%)	59.91% (8.86%)	9.28% (9.85%)
3	2	Test	51	60.72% (10.40%)	50.02% (9.97%)	10.7% (11.9%)
3	3	Training	51	68.13% (6.97%)	61.64% (8.21%)	6.49% (7.56%)
3	4	Test	51	59.63% (10.97%)	52.3% (8.98%)	7.34% (12.46%)
3	5	Training	51	70.30% (5.66%)	63.07% (8.81%)	7.23% (8.32%)
3	6	Test	51	60.72% (11.56%)	51.95% (9.38%)	8.77% (13.4%)
3	7	Training	51	70.64% (5.98%)	65.74% (9.46%)	4.89% (9.58%)
3	8	Test	51	57.82% (9.51%)	52.29% (10.38%)	5.52% (12.6%)

*Note*: SSPA = Sequence-Specific Performance Advantage. The Block length is 540 trials.

**Table A2:** Accuracy of trained and untrained sequences for each training and test block in Experiment 4.

Day	Block	Phase	*n*	Trained Sequence M (SD)	Untrained Sequence M (SD)	SSPA M (SD)
Day 1	1	Training	165	71.68% (10.92%)	66.50% (12.3%)	5.18% (8.47%)
Day 1	2	Test	165	70.18% (14.31%)	63.33% (13.81%)	6.85% (10.39%)
Day 1	3	Training	165	74.53% (9.68%)	69.58% (11.52%)	4.95% (9.44%)
Day 1	4	Test	165	70.14% (14.02%)	63.29% (14.56%)	6.85% (10.9%)
Day 1	5	Training	165	75.19% (9.83%)	70.52% (11.15%)	4.67% (8.98%)
Day 1	6	Test	165	69.55% (14.83%)	64.04% (13.56%)	5.51% (11.07%)
Day 1	7	Training	165	75.86% (9.72%)	70.64% (9.51%)	5.22% (8.14%)
Day 1	8	Test	164	69.52% (14.13%)	63.39% (13.5%)	6.13% (10.17%)
Day 2	1	Training	165	78.79% (8.26%)	71.52% (10.99%)	7.28% (10.04%)
Day 2	2	Test	165	71.32% (11.84%)	64.92% (12.69%)	6.40% (9.58%)
Day 2	3	Training	165	78.31% (8.62%)	71.51% (10.35%)	6.81% (8.36%)
Day 2	4	Test	165	71.52% (11.78%)	65.41% (12.76%)	6.11% (8.66%)
Day 2	5	Training	165	78.36% (8.04%)	72.64% (10.75%)	5.72% (8.97%)
Day 2	6	Test	165	70.04% (12.80%)	65.62% (12.72%)	4.41% (9.99%)
Day 2	7	Training	165	78.48% (8.69%)	72.93% (10.1%)	5.56% (8.73%)
Day 2	8	Test	163	70.39% (13.74%)	66.3% (11.63%)	4.08% (9.13%)
Day 3	1	Training	165	80.77% (7.97%)	73.84% (9.76%)	6.92% (9.04%)
Day 3	2	Test	165	72.95% (11.96%)	66.28% (11.82%)	6.67% (8.94%)
Day 3	3	Training	165	80.52% (7.73%)	74.95% (9.33%)	5.57% (8.49%)
Day 3	4	Test	165	72.00% (11.03%)	66.64% (11.94%)	5.37% (10.04%)
Day 3	5	Training	165	79.96% (7.69%)	76.11% (9.61%)	3.86% (8.2%)
Day 3	6	Test	165	72.40% (11.51%)	67.51% (11.43%)	4.90% (8.91%)
Day 3	7	Training	165	80.65% (7.67%)	76.51% (9.93%)	4.14% (7.87%)
Day 3	8	Test	164	71.70% (11.18%)	67.50% (11.55%)	4.20% (8.33%)

*Note:* SSPA = Sequence-Specific Performance Advantage. The Block length is 540 trials.

## Appendix D

**Table A3:** Descriptive statistics of the cognitive battery scores in Experiment 4.

Measure	*n*	*Mean*	*SD*
BFAS	165	39.3	6.8
DSS	165	38.6	9.2
LN	164	5.4	2.3
LS	165	5.7	1.2
MR	165	6.4	2.7
NEO-PI-R	165	133.5	17.8
OSPAN	161	46.4	11.7
SOIP	164	22.5	5.1
SeVi	165	4.5	1.2

*Note:* BFAS = Openness on Big Five Aspect Scales, DSS = Digit Symbol Substitution, LN = Letter and Number Series, LS = List Sorting, MR = Matrix Reasoning, NEO-PI-R = Openness on Revised NEO Personality Inventory, OSPAN = Operation Span, SOIP = Speed of Information Processing, SeVi = Sequential Visuospatial.
